# Emerging Role of Non-Coding RNAs in Regulation of T-Lymphocyte Function

**DOI:** 10.3389/fimmu.2021.756042

**Published:** 2021-11-04

**Authors:** Mohammad Taheri, Dominik A. Barth, Julia Kargl, Omidvar Rezaei, Soudeh Ghafouri-Fard, Martin Pichler

**Affiliations:** ^1^Skull Base Research Center, Loghman Hakim Hospital, Shahid Beheshti University of Medical Sciences, Tehran, Iran; ^2^Division of Oncology, Department of Internal Medicine, Medical University of Graz, Graz, Austria; ^3^Otto Loewi Research Center, Division of Pharmacology, Medical University of Graz, Graz, Austria; ^4^Department of Medical Genetics, School of Medicine, Shahid Beheshti University of Medical Sciences, Tehran, Iran; ^5^Research Unit of Non-Coding RNAs and Genome Editing in Cancer, Division of Clinical Oncology, Department of Internal Medicine, Comprehensive Cancer Center Graz, Medical University of Graz, Graz, Austria; ^6^Department of Experimental Therapeutics, The University of Texas MD Anderson Cancer Center, Houston, TX, United States

**Keywords:** miRNA, lncRNA, circRNA, T cell, cancer, autoimmune

## Abstract

T-lymphocytes (T cells) play a major role in adaptive immunity and current immune checkpoint inhibitor-based cancer treatments. The regulation of their function is complex, and in addition to cytokines, receptors and transcription factors, several non-coding RNAs (ncRNAs) have been shown to affect differentiation and function of T cells. Among these non-coding RNAs, certain small microRNAs (miRNAs) including miR-15a/16-1, miR-125b-5p, miR-99a-5p, miR-128-3p, let-7 family, miR-210, miR-182-5p, miR-181, miR-155 and miR-10a have been well recognized. Meanwhile, IFNG-AS1, lnc-ITSN1-2, lncRNA-CD160, NEAT1, MEG3, GAS5, NKILA, lnc-EGFR and PVT1 are among long non-coding RNAs (lncRNAs) that efficiently influence the function of T cells. Recent studies have underscored the effects of a number of circular RNAs, namely circ_0001806, hsa_circ_0045272, hsa_circ_0012919, hsa_circ_0005519 and circHIPK3 in the modulation of T-cell apoptosis, differentiation and secretion of cytokines. This review summarizes the latest news and regulatory roles of these ncRNAs on the function of T cells, with widespread implications on the pathophysiology of autoimmune disorders and cancer.

## Introduction

T-lymphocytes (T-cells) play a central role in adaptive immunity and are involved in the pathogenesis of immune-related disorders and cancer, thus several therapeutic strategies have been developed to stimulate their effector functions (1). During the process of maturation in the thymus, T cells express T cell receptors (TCR) on their surface. Moreover, they can express either CD8 or CD4 glycoproteins, thus being categorized as glycoprotein on their surface and are called CD8+ (cytotoxic) or CD4+ cells (helper) T cells (2). Based on the distinctive cytokine profiles, T helper (Th) cells can be categorized to Th1, Th2, Th9, Th17, Th22, regulatory T cells (Tregs), and follicular helper T cells (Tfhs) subtypes (3). Each cell type can be recognized by epigenetic and genetic signatures. For instance, Treg cells are described by over-expression of the FOXP3 transcription factor (4) Demethylation of the intronic conserved non-coding sequence 2 is required for maintenance of FOXP3 expression and regulation of stability of Tregs upon re-exposure to cytokines (5). In Tregs, this intronic sequence acts a sensor for IL-2 and STAT5 (5). The expression of a number of transcription factors has been shown to be altered in CD8+ T cells during clearing an Bacterial or Viral infection (6). Notably, it is possible to predict the potential of these cells to make memory cells based on gene signatures (6). For instance, expressions of Bcl-2 and Cdh-1 have been shown to be surged in the memory subset of CD8+ T cells (6). In addition, chromatin configurations have been found to influence the function of T cells (6). Non-coding RNAs (ncRNAs) carry a regulatory function in several biological processes including implications in immune checkpoint inhibitor treatment (7). Recent studies have highlighted the impact of different classes of non-coding RNAs in T cell functions. In this review, we highlight the function of microRNAs (miRNAs), long non-coding RNAs (lncRNAs) and circular RNAs (circRNAs) in regulation of T cells. These three classes of ncRNAs have regulatory effects on expression of mRNA coding genes. In fact, lncRNAs and circRNAs can sequester miRNAs and decrease availability of miRNAs. Since miRNAs can inhibit expression of target mRNAs, the sequestering effects of circRNAs and lncRNAs on miRNAs release miRNA targets from inhibitory effects of miRNAs (8).

## miRNAs and T Cell Regulation

miRNAs are about 22 nucleotides in length and regulate expression of their target transcripts mostly through binding to their 3’ UTR (9, 10). These small molecules are generated in the forms of precursors by RNA polymerases II and III. The mature miRNA is generated through a series of cleavage events in the nucleus and cytoplasm (9). Given their various regulatory functions, miRNAs are important players in the regulation of several physiologic and pathophysiologic processes (11). As for the regulation of T-cell differentiation, several examples of important miRNAs have been reported. For instance, miR-15/16 hampers T cell cycle, their survival and differentiation to memory T cells. Experiments in miR-15/16 deficient T cells have shown that these miRNAs directly inhibit the expression of a broad network of genes contributing in the regulation of cell cycle progression, survival, and development of memory cells (12). Another study has shown miR-15a/16-1 silencing in CD4+ T cells leads to the production of higher levels of IL-22, while up-regulation of miR-15a/16-1 results in down-regulation of the IL-22 expression through suppression of the aryl hydrocarbon receptor. miR-15a/16-1 silenced CD4+ T cells were superior to wild-type CD4+ T cells in terms of tissue repair capacity because of their higher capability in production of IL-22. Furthermore, IL-22 has been shown to decrease miR-15a/16-1 levels through activation of phosphorylated STAT3-c-myc signaling (13).

A high throughput miRNA profiling in human peripheral γδ T cells of healthy subjects has led to identification of 14 differentially expressed miRNAs between αβ and γδ T cells. While miR-150-5p, miR-450a-5p, miR-193b-3p, miR-365a-3p, miR-31-5p, miR-125b-5p and miR-99a-5p have been up-regulated in γδ T cells, miR-34a-5p, miR-16-5p, miR-15b-5p, miR-24-3p, miR-22-3p, miR-22-5p and miR-9-5p have had the opposite trend (14). Notably, miR-125b-5p and miR-99a-5p have been found to attenuate the activity of γδ T cells and decrease their cytotoxic effects against tumor cells. Up-regulation of miR-125b-5p or miR-99a-5p in γδ T cells was shown to suppress the activity of γδ T cells and induced their apoptosis. Moreover, miR-125b-5p silencing has increased cytotoxic effects of γδ T cells against tumor cells through enhancing the production of IFN-γ and TNF-α (14). Overexpression of miR-125b has also promoted Treg cells differentiation and suppressed Th17 cell differentiation (15). In addition, miR-125a, a miRNA which has only recently been reported to be involved in myelogenous leukemogenesis (16), could inhibit production of proinflammatory cytokines in CD4+ T cells and Th1/Th17 cell differentiation by targeting ETS-1 (17).

Let-7 family miRNAs are also involved in the regulation of T cells functions. *In vivo* experiments demonstrated that, let-7g-5p may attenuate the frequency of Th17 cells of rheumatoid arthritis (RA) and block Th17 differentiation (18). Let-7f-5p inhibits Th17 differentiation through targeting STAT3. This miRNA has been found to be downregulated in CD4+ T cells of patients with multiple sclerosis (MS) (19). Finally, let-7d-3p regulates the expression of IL-17 in CD4 + T cells by targeting AKT1 and modulation of AKT1/mTOR signaling pathway (20).

miR-210 is another miRNA whose deletion enhances T cell differentiation and Th17 polarization under hypoxic situation through modulation of HIF-1α expression (21). Expression of this miRNA has also been found to be over-expressed in both psoriasis patients and psoriasis animal models where it stimulates Th17 and Th1 cell differentiation but suppresses Th2 differentiation *via* inhibiting expressions of STAT6 and LYN. Ablation of miR-210 in animals and intradermal injection of miR-210 antagonist has reversed the immune imbalance and blocked the development of psoriasis-like inflammatory response in an animal model of psoriasis. TGF-β and IL-23 have been shown to increase the expression of miR-210 through the induction of HIF-1α, and subsequent recruitment of P300 and enhancement of histone H3 acetylation in miR-210 promoter (22).

**Figure 1:**
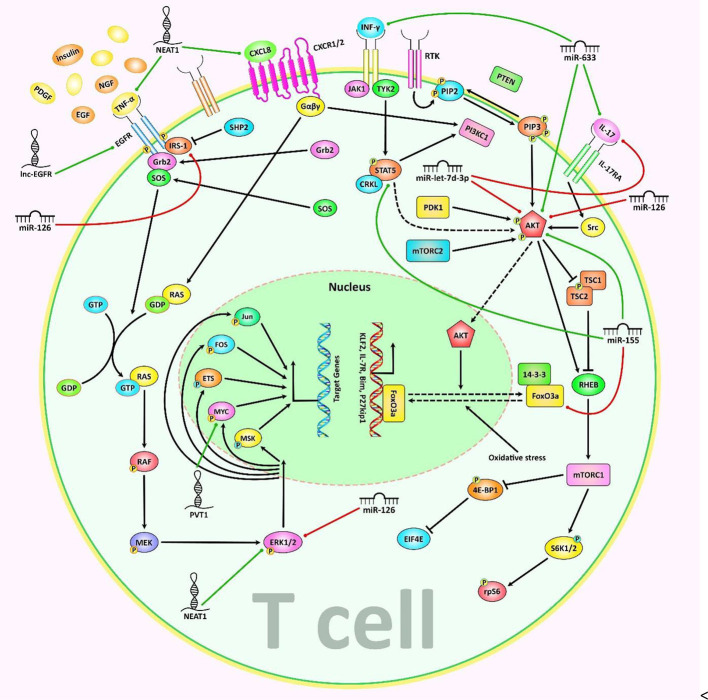
A schematic representation of the role of various non-coding RNAs in modulating the differentiation of T cells *via* the PI3K/Akt/mTOR and MAPK/ERK signaling cascades. a) The MAPK/ERK pathway can be triggered *via* several growth factors including PDGF, EGF, NGF, and insulin. Upon receptor dimerization, activation of its tyrosine kinase module could be triggered, subsequently recruiting Grb2 and SOSto the phosphorylated domain, thus creating the Grb2-SOS complex. Furthermore, the GTP binding protein RAS interacts with the Grb-2-SOS complex that in turn leads to the activation of RAS. Activated GTP-bound RAS plays an effective role in upregulating the phosphorylation of MEK1/2 (MAPKK), which then phosphorylates ERK1/2 (MAPK). Eventually, ERK is transferred to the nucleus where it triggers the activation of various target genes involved in a variety of cellular processes (26–29). b) The PI3K/Akt/mTOR signaling is activated by a subset of growth factors such as PI3KCI, which phosphorylates PIP2 to PIP3. PIP3 has an important role in recruiting AKT which gets activated through double phosphorylation (via PDK1 and mTORC2). In addition, activated AKT suppresses TSC2 through phosphorylation. Inactive TSC1/2 complex is able to bind RHEB, which eventually triggers the activation of mTORC1. The mTORC1 has a significant impact on many downstream proteins, such as S6K1/2 and 4EBP1 (30, 31). Besides, exposure to IL-17 results in receptor-mediated activation of Src, MAPKs, and PI3K/Akt signaling cascades (32). Moreover, subsequent to JAK activation, CRKL is phosphorylated by TYK2 that could result in CRKL complexation with STAT5. STATs in turn interacts with individual mediators of the PI3K/AKT signaling cascade (33). Accumulating finding has demonstrated that miR-let-7d-3p *via* directly suppressing AKT1 could regulate expression level of IL-17 in CD4+ T cells through the AKT1/mTOR signaling pathway (20). In addition, another research has authenticated that overexpression of lncRNA NEAT1 could promote the expression levels of CXCL8 and TNF-α in Sjögren’s syndrome *via* positively regulating MAPK signaling (34). Green arrows indicate upregulation of target genes modulated *via* ncRNAs (lncRNAs, and miRNAs), red arrows depict inhibition by these ncRNAs. All the information regarding the role of these ncRNAs in modulating T call differentiation can be seen in and.

miR-181c has been shown to enhance Th17 differentiation and promote autoimmunity through targeting Smad7 and modulating TGF-β pathway and IL-2 expression (13). Overexpression of miR-181c has suppressed activation of T cell, impaired cytoskeleton arrangement in T cells by targeting BRK1 (23). Meanwhile, miR-181a has been reported to restrict IFN-γ production by targeting Id2 so regulating IFN-γ-mediated CD8+ T cell responses mediated by (24). This miRNA also promotes expression of TGF-β and IL-10 and inhibits function of Tregs through modulating the PI3K/Akt pathway (25). **Figure 1** illustrates the role of various ncRNAs in regulating the differentiation of T cells *via* the PI3K/Akt/mTOR and MAPK/ERK signaling pathways. **Table 1** summarizes the impact of miRNAs on regulation of function of T cells.

**Table 1:** miRNAs and T cell regulation.

microRNA	Expression pattern	Disease	Sample	Cell line	Interaction	Signaling pathway	Function	Reference
*miR-15/16*	–	–	miR-15/16 deficient mouse model	CD4(+) T cells obtained from mice	–	–	Constrains formation of memory T cells and confines T cell survival and cell cycle through modulating complex network of their target genes implicated in cell cycle and survival	(12)
*miR-15a/16-1*	–	–	C57BL/6 mice	Naïve CD4+ T cells	AHR	–	Decreasing IL-22 secretion of CD4+ T cells through targeting AHR	(13)
*miR-125b-5p* *miR-99a-5p*	Downregulated (in γδ T cells compared with αβ T cells)	–	Peripheral blood obtained from 21 healthy donors	αβ T cells and γδ T cells purified from peripheral blood	–	–	upregulation inhibits activation of γδ T cells and cytotoxicity to tumor cells by decreasing secretion of IFN-γ and TNF-α.	(14)
*miR-125b*	Downregulated (in in PBMCs and CD4+ T cells of patients)	Juvenile idiopathic arthritis (JIA)	Peripheral blood obtained from 16 JIA patients and 22 healthy volunteers, 24 male DBA/1J mice	CD4+ T cells	–	–	overexpression promotes Treg cells differentiation and suppresses Th17 cell differentiation.	(15)
*miR-125a*	Downregulated (in PBMC of IBD patients)	Inflammatory bowel diseases (IBD)	Blood samples from 106 IBD patients and 16 healthy controls, Female C57BL/6 mice	CD4+ T cells	ETS-1↑	–	Inhibited production of proinflammatory cytokines in CD4+ T cells and Th1/Th17 cell differentiation by targeting ETS-1	(17)
*miR-128-3p*	Upregulated (in T cells RA patients)	Rheumatoid arthritis (RA)	Blood samples from 20 patients with RA and 20 healthy subjects, C57BL/6 mice	Patient derived T cells	TNFAIP3	NF-κB signaling pathway	silencing represses activation of T cells by upregulating TNFAIP3 and inhibiting NF-κB signaling pathway	(35)
*let-7g-5p*	Downregulated (in plasma of RA patients)	Rheumatoid arthritis (RA)	Plasma samples from RA patients and healthy controls, C57BL/6 mice, DBA 1/J mice	CD4+ T cells	–	–	Upregulation attenuates Th17 frequency in RA mouse model and blockes Th17 differentiation.	(18)
*let-7f-5p*	Downregulated (in CD4+ T cells of patients with MS)	Multiple sclerosis (MS)	Blood samples from 16 RRMS patients and 16 healthy controls, Female C57BL/6J mice	CD4+ T cell	STAT3↑	–	Overexpression inhibits Th17 differentiation through targeting STAT3.	(19)
*miR-let-7d-3p*	–	Primary Sjögren’s syndrome (pSS)	Blood samples from pSS patients and healthy controls	CD4+ T cells	AKT1	AKT1/mTOR signaling pathway	Regulates expression of IL-17 in CD4 + T cells by targeting AKT1 and modulation of AKT1/mTOR signaling pathway	(20)
*miR-183* *miR-96*	Upregulated Upregulated (in patients’ T cells and activated T cells from controls)	Graves’ orbitopathy (GO)	Blood samples from patients with GO and normal subjects, TCR-HA/Thy.1.1 transgenic mice, INS-HA/Rag2KO transgenic mice and BALB/c mice	CD4(+) T cells from human blood samples and mice	EGR-1	–	overexpression was associated with lowered EGR-1 expression and augmented proliferation while their downregulation had reverse effects	(36)
*miR-210*	Upregulated (in activated T cells)	Chronic colitis	Mir210 conditional knockout mice	Naive T cells, TH17 cells	HIF-1α	–	deletion potentiates T cell differentiation and TH17 polarization by modulation of HIF-1α expression	(21)
*miR-210*	Upregulated (in psoriasis patients)	Psoriasis	Blood samples and skin tissues specimens from 63 psoriasis patients and 80 normal volunteers, C57BL/6J and BALB/c mice	CD4+ T	STAT6, LYN	–	Enhances Th1 and Th17 differentiation and represses Th2 differentiation by targeting STAT6 and LYN	(22)
*miR-182-5p*	Downregulated (in Th17 cells of EAU mice)	Uveitis	Blood samples from 15 patients with Behçet’s disease with uveitis, 15 patients with active sympathetic ophthalmia with uveitis and 15 healthy subjects, C57BL/6 mice	CD4+ T-cells, EL4 murine T cell line	TAF15	STAT3 signaling pathway	overexpression inhibits Th17 development and lowers diseased severity in experimental autoimmune uveitis by targeting TAF15 and modulating STAT3 pathway	(37)
*miR-182*	Upregulated (in CD4+ T cells of RRMS patients)	Relapse and remitting multiple sclerosis (RRMS)	Blood samples from RRMS patients and healthy controls, female C57BL/6 mice	CD4+ T cells	HIF-1α	–	Its overexpression led to promoted differentiation of naïve T cells to Th1 and Th17 through targeting HIF-1α and rising IFN-γ expression.	(38)
*miR-181c*	–	Multiple sclerosis (MS)	Female C57BL/6 mice	CD4+ CD62L+ T helper cells	Smad7	TGF-β signaling pathway	Enhanced Th17 differentiation and promoted autoimmunity through targeting Smad7 and modulating TGF-β pathway and IL-2 expression	(13)
*miR-181c*	Downregulated (in activated T cells)	–	–	MCF7, HeLa, CD3+ T cells, (Jurkat T cells	BRK1	–	Its overexpression suppressed activation of T cell, impaired cytoskeleton arrangement in T cells by targeting BRK1.	(23)
*miR-181a*	–	–	C57BL/6J mice	CD8+ T cell	Id2	–	Restricted IFN-γ production by targeting Id2 so regulated CD8+ T cell responses mediated by IFN-γ	(24)
*miR-181a*	–	Allergic rhinitis (AR)	C57BL/6 mice	CD4+ T cells, Treg cells	–	PI3K/Akt pathway	Promoted expression of TGF-β and IL-10 and inhibited function of Tregs through modulating PI3K/Akt pathway	(25)
*miR-202-5P*	Upregulated (in PBMCs, Tregs, and CD4+ T cells of AR patients)	Allergic rhinitis (AR)	Blood samples from 30 AR cases and 10 normal controls	Tregs cells, CD4+ T cells	MATN2	–	Repressed differentiation of Tregs by targeting MATN2	(39)
*miR-155*	–	Allergic rhinitis (AR)	C57BL/6 mice	CD4+ T cells, Treg cells	–	SOCS1 and SIRT1 signaling pathway	Elevated proliferation of Treg cells by modulating SOCS1 and SIRT1 signaling pathway but no influence on T cell function suppression	(25)
*miR-155*	Upregulated (in donor T cells in aGVHD patients)	Acute graft versus host disease (aGVHD)	C57BL/6 (B6, H2^b^), C57BL/6-Tg(CAG-EGFP)1Osb/J (B6 GFP, H2^b^), Cg-miR-155tm1.1Rsky/j (miR-155−/−, H2^b^), B6D2F1 (F1, H2^b/d^), BALB/c (H2^d^), and C3.SW-H2^b^/SnJ (H2^b^)	–	–	–	Its expression in CD8+ and CD4+ T cells is necessary for pathogenesis of aGVHD through regulation of migration, expansion and effector function of T cell	(40)
*miR-155*	–	Viral infection	C57BL/6, MiR-155−/−, wild-type (WT) and ovalbumin-specific Tcrα/Tcrβ transgenic (OTII) mice	CD4+ T	–	–	Is implicated in regulation of proliferation, activation and cytokine production of CD4+ T	(41)
*miR-155*	–	Vitiligo	Blood samples from one vitiligo patient and one healthy donor	naïve T and CD8+ T cells	–	–	Its overexpression decreased proliferation of CD8+ T cells and enhanced Treg percentage	(42)
*miR-155*	–	Glioma	C57BL/6 mice	GL261, T cell	FoxO3a	Akt and Stat5 signaling pathway	Its upregulation promoted proliferation and activation of T cells and increased their cytotoxicity by targeting FoxO3a and modulating Akt and Stat5 signaling pathway	(43)
*miR-149-3p*	Downregulated (in CD8+ T cells overexpressing PD-1)	Breast cancer	Female BALB/c mice	4T1, CD8+ T cell	–	–	Its overexpression reduced T cell apoptosis and expression of T cell inhibitor receptors, also promoted activation of T cells	(39)
*miR-143*	Upregulated (in naïve and memory T cells compared with effector T cells)	Esophageal squamous cell carcinoma (ESCC)	13 tumor tissues and adjacent normal tissues from 13 ESCC patients and blood samples from 10 healthy donors	CD8+ T cell, HER2-CAR T cells	Glut-1	–	Its upregulation promoted differentiation of CD8+ T cell to memory T cells, raised T cell cytotoxicity and decreased apoptosis by targeting Glut-1 and regulation of metabolism	(44)
*miR-17-92*	–	Chronic graft-versus-host disease (cGVHD)	miR-17-92 conditional knockout (KO) mice	CD4+ T	–	–	Increased differentiation of Th1 and Th17 cells, elevated production of follicular Th cells and associated with scleroderma and bronchiolitis	(45)
*miR-10a*	–	–	3 Adipose Tissue healthy subjects, female C57BL/6 mice	Naïve CD4+ T cell, adipose tissue derived mesenchymal stem cells (AD-MSCs)	–	–	Transfection with miR-10a-loaded exosomes derived from AD-MSCs elevated expression of RORγt and Foxp3 and reduced expression of T-bet and led to differentiation of naive T cells to Th17 and Treg	(46)
*miR-10a-3p*	Downregulated (in PBMC of LN patients)	Lupus nephritis (LN)	Blood samples from 94 LN patients and 38 healthy subjects	–	REG3A↑	JAK2/STAT3 pathway	Its upregulation enhanced Treg cells and lessened Th17/Treg ratio and alleviated renal function by targeting REG3A	(47)
*miR-633*	Downregulated (in CD4+T cells of SLE patients)	Systemic lupus erythematosus (SLE)	Blood samples from 20 SLE patients and 19 healthy controls	CD4+T cells, Jurkat cells	AKT1↑	AKT/mTOR pathway	Its downregulation increased IL-17, and IFN-γ production and activated AKT/mTOR pathway in CD4+T cells through modulating AKT1	(48)
*miR-142-3p*	Upregulated (in CD4+ T cells of T1D patients)	Type 1 diabetes (T1D)	Blood samples form T1D patients, CBy.PL(B6)-Thy1^a^/ScrJ (CD90.1 BALB/c), Balb/cByJ (CD90.2 BALB/c), Balb/c.Cg-Foxp3tm2Tch/J (BALB/c Foxp3GFP), and NOD/ShiLtJ mice, NOD.Cg-Prkdc^scid^ H2-Ab1^tm1Gru^ Il2rg^tm1Wjl^ Tg(HLA-DQA1,HLA-DQB1) 1Dv//Sz mice	CD4+ T cells	Tet2	–	Inhibited differentiation of Treg cells and decreased stability of Tregs by targeting Tet2 and its depletion collapsed islet autoimmunity in mouse models of diabetes	(49)
*miR-142-3p*	–	Acute graft versus host disease (GVHD)	Blood samples form volunteer donors, NOD/SCID/mice	Thymic-derived regulatory T cell (tTreg) (CD4 + CD25 + CD127-tTreg)	ATG16L1	–	Its knockdown enhances survival and proliferation of tTregs by upregulating expression of ATG16L1 and modulating autophagy	(50)
*miR-142-3p*	–	–	Blood samples from healthy volunteers, NOD CRISPR Prkdc Il2r gamma (NCG) mice	Naïve CD4+CD45RA+ T cells	KDM6A	–	Its knockdown improved regulatory function and expression of cytokines and suppressed apoptosis in iTregs by upregulating KDM6A	(51)
*miR-26b-5p*	Downregulated (in HCC tissues and CD4+ and CD8+ T cells)	Hepatocellular carcinoma (HCC)	42 HCC tissues and ANTs, SPF C57BL/6 and nude mice	CD4+ and CD8+ T cells	PIM-2	–	Its overexpression improved cytokine secretion of CD4+ and CD8+ T cells by targeting PIM-2	(48)
*miR-34a*	–	–	Blood samples from healthy donors	CD4+ and CD8+ T cells	PLCG1, CD3E, PIK3CB, TAB2, NFκBIA	NF-κB signaling pathway	Its overexpression suppressed expression of its target genes in CD4+ and CD8+ T cells and lowered cytotoxic ability of T cells through modulating NF-κB signaling	(52)
*miR-34a*	Downregulated (in tumor-infiltrating T cells)	Gastric cancer (GC)	Blood samples from 73 GC patients and 58 healthy controls	Jurkat cell	LDHA	–	Its overexpression decreased lactate level in T cells and increased IFN-γ expression through targeting LDHA	(53)
*miR-140-5p*	Downregulated (in encephalomyelitic CD4+T cells)	Experimental autoimmune encephalomyelitis (EAE)	Female C57BL/6 mice	CD4+T cells	–	–	Its upregulation constrained Th1 differentiation through regulating methylation of STAT1 and Tbx and modulation of mitochondrial respiration	(17)
*miR-130a−3p*	Downregulated (T cells AS patients)	Ankylosing spondylitis (AS)	Blood samples from 30 HLA-B27-positive AS patients and 30 HLA-B27-negative healthy controls	Jurkat T cells	HOXB1	–	Its overexpression resulted in increased proliferation and decreased apoptosis rate in T cells through targeting HOXB1	(54)
*miR-126*	–	Acute autoimmune colitis	Friend leukaemia virus B (FVB)/N miR‐126 knock down mice	CD4+ T cells	IRS-1	AKT and NF-κB pathways	Its knockdown was associated with elevated proliferation and activation of CD4+ T cells and augmented expression of IFN-γ	(55)
*miR-425*	Upregulated (in PBMC of IBD patients)	Inflammatory bowel disease (IBD)	Blood samples from 124 IBD patients and healthy controls, Female BALB/c mice	CD4+ T cells	Foxo1↓	–	Promoted Th17 differentiation from CD4+ T cells through targeting Foxo1	(56)
*miR-219a-5p*	Downregulated (in CD4+ T cells of IBD patients)	Inflammatory bowel disease (IBD)	Blood samples from 33 IBD patients and 23 healthy individuals, female BALB/c mice	CD4+ T cells	ETV5↑	–	Its overexpression inhibited Th1/Th17 cell differentiation by targeting ETV5 and regulating phosphorylation of STAT3 and STAT4	(57)
*miR-22*	Upregulated (in intestinal tissues and CD4+ T cells of IBD patients)	Inflammatory bowel disease (IBD)	Intestinal tissues and blood samples from 99 IBD patients, 15 intestinal tissues from patients with colonic polyps and 20 blood samples from healthy controls	CD4+ T cells	HDAC4	–	Elevated Th17 differentiation and inflammatory cytokines production by targeting HDAC4	(58)
*miR-21-5p*	Downregulated (in PBMC of vitiligo patients)	Vitiligo	Blood samples from 15 vitiligo patients and 15 healthy controls	CD4+ T cells	STAT3↓	–	Its overexpression increased Treg cells proportion and decreased effector T cells (Teff), so balanced Treg/Teff ratio by targeting STAT3	(59)
*miR-223-3p*	Upregulated (in Th17 cells)	Experimental autoimmune uveitis	Female C57BL/6	CD4+ T cells	FOXO3	–	Induced autoreactive Th17 responses by targeting FOXO3 and modulation of IL-23 receptor expression	(60)
*miR-669b-3p*	–	–	C57BL/6 (H-2b) and BALB/c (H-2d) mice	CD4+ T cells	–	–	Increased proliferation of CD4+ T cells and restrained apoptosis of these cells by negative regulation of IDO	(61)
*miR-146a*	Upregulated (in CD27- γδ T cells)	–	C57BL/6J and CD45.1 mice, Rag2−/− mice, Il17a-GFP knock-in mice, miR-146a−/− mice, Nod1−/− and Atf2−/− mice	CD27- γδ T cells and CD27+ γδ T cells, CD4+ T cells	NOD1		Decreased IFN-γ production and restricted functional plasticity of γδ T cells through targeting NOD1	(62)
*miR-29b*	Upregulated (in CD4+ T cells of OLP patients)	Oral lichen planus (OLP)	Blood samples form 18 OLP patients and 18 age- and gender-matched controls	CD4+ T cells	–	–	Inhibited IFN-γ expression and secretion in CD4+ T cells, also suppressed expression of DNMT1 induced global DNA hypomethylation in CD4+ T cells to Th1 responses	(63)
*miR-31*	Upregulated (in peripheral blood of CHD patients)	Coronary heart disease (CHD)	Blood samples from 56 CHD patients and 47 non-CHD individuals	CD4+ T cells	Bach2	–	Increased Th22 differentiation by targeting Bach2	(64)
*miR-653*	Downregulated (in thymic tissues of MG mice)	Myasthenia gravis (MG)	Thymic tissues from 42 MG patients, BALB/c male nude mice	Thymocytes obtained from thymic tissues	TRIM9	–	Its overexpression decreased viability of thymocytes and induced cell cycle arrest and apoptosis in these cells by targeting TRIM9	(65)
*miR-192*	Downregulated (in plasma and CD4+ T cells of asthma patients)	Childhood asthma	Blood samples from 18 children with childhood asthma and 15 healthy children	CD4+ T cells	CXCR5	–	Its overexpression impeded activation of T follicular helper cells by targeting CXCR5	(66)
*miR-23a-3p*	Downregulated (in CD4+ T cells of GD patients)	Graves’ disease (GD)	Blood samples from 32 GD patients and 20 healthy individuals, female Balb/c mice	CD4+ T cell, 293T	SIRT1	–	Its overexpression enhanced Treg frequency and improved function of Tregs by targeting SIRT1 and modulating FOXP3 expression and acetylation	(67)
*miR-133a/133b*	Upregulated (in PBMC of IgAN patients)	IgA nephropathy (IgAN)	Blood samples form 20 IgAN patients and heakthy controls	CD4+ T cells	FOXP3	–	Inhibited Treg differentiation and decreased Treg frequency by downregulating FOXP3	(68)

## LncRNAs and T Cell Regulation

LncRNAs are typically longer than 200 nucleotides and may also be several kilobases long (69). They exert diverse effects on chromatin structure, transcription of genes and post-transcriptional regulation of gene expression (70). These effects are exerted through both chromatin-based mechanisms and the interaction with other types of transcripts. Moreover, by serving as decoy, scaffold, and enhancers, lncRNAs influence genes expressions though various mechanisms (71). Several lncRNAs have been found to influence the function of T cells. For instance, IFNG-AS1 is up-regulated in the intestinal tissue of patients with active inflammatory bowel disease (IBD). Specific over-expression of IFNG-AS1 in T cells has led to significant enhancement of inflammatory cytokines, while attenuation of production of anti-inflammatory cytokines. Media from IFNG-AS1-overexpressing T cells has induced a potent inflammatory response in primary human peripheral blood mononuclear cells (PBMCs) (72). Lnc-ITSN1-2 is another lncRNA that affect T cells differentiation. This lncRNA has been shown to increased proliferation and activation of CD4+ T Cells and promote their differentiation to Th1/Th17 through targeting miR-125a and upregulating IL-23R (73).

The regulatory role of NEAT1 on T cells functions has been validated in different contexts, including sepsis, primary Sjögren’s syndrome, RA and hepatocellular carcinoma (HCC) (74, 75). Downregulation of NEAT1 has restricted immune response in mouse model of sepsis and induced T cell apoptosis through modulating miR-125/MCEMP1 axis (76). This lncRNA has been shown to promote expression of CXCL8 and TNF-α and activate MAPK signaling pathway. NEAT1 expression has been up-regulated in CD4+ and CD8+ T cells of patients with primary Sjögren’s syndrome (34). Similarly, this lncRNA has been found to be up-regulated in peripheral blood mononuclear cells of RA patients. Its silencing has led to inhibited differentiation of Th17 cells from CD4+ T cells by downregulating STAT3 through modulating its ubiquitination (77). Finally, NEAT1 has been found to be up-regulated in PBMCs of HCC patients parallel with up-regulation of Tim-3. NEAT1 silencing has blocked apoptosis of CD8+T cells and increased their cytolysis function. Further, NEAT1 has been shown to exert such effects through miR-155/Tim-3 pathway. Taken together, NEAT1 has been suggested as an important target for enhancing the efficiency of immunotherapy (78).

**Figure 2:**
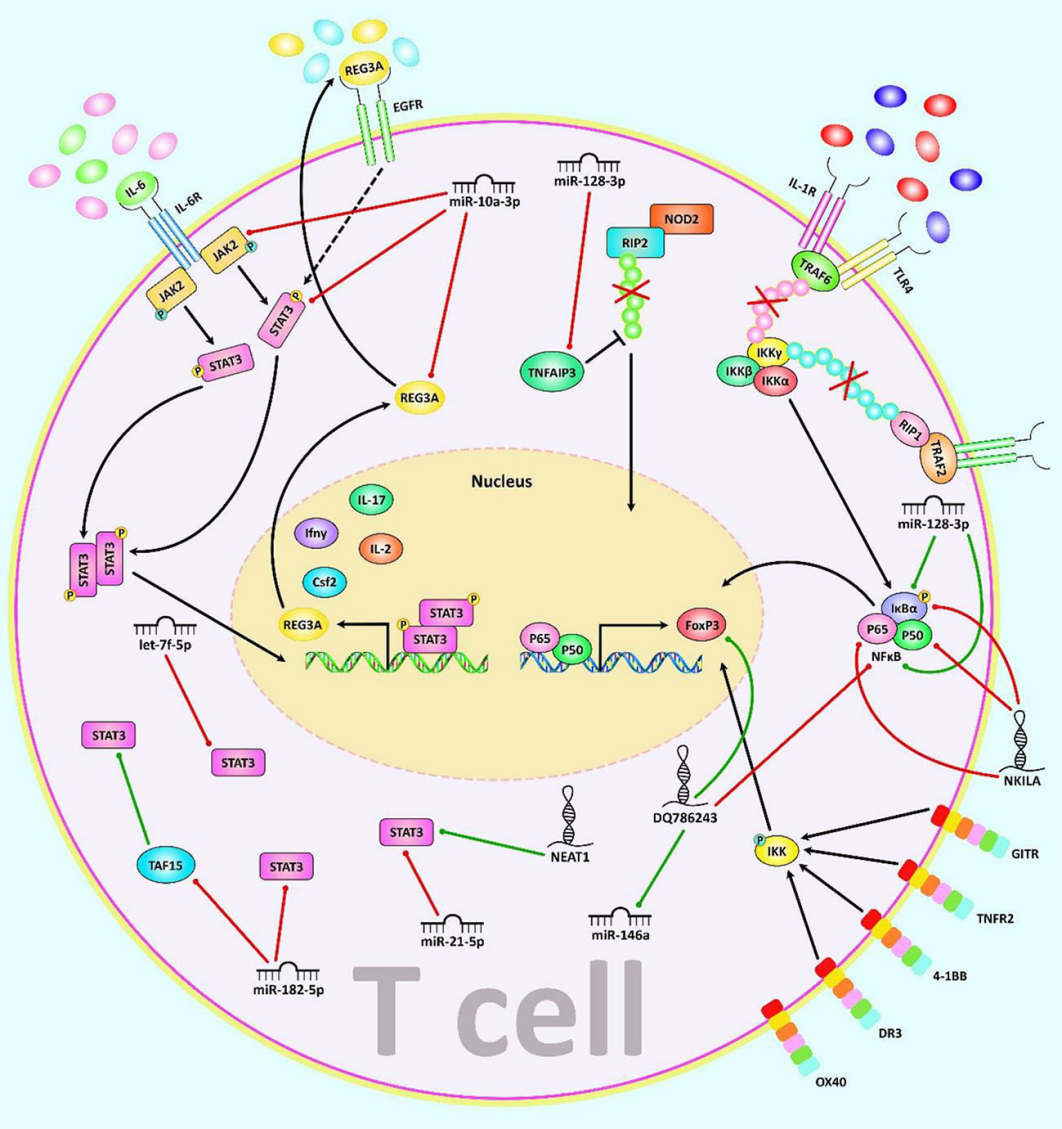
A schematic illustration of the role of various noncoding-RNAs in modulating the JAK2/STAT3 and NF-κB signaling pathway as major regulators of T cell function. a) In JAK/STAT pathway, JAKs bind to the receptor, and upon multimerization, upregulation of JAK proteins is mediated *via* trans-phosphorylation. Consequently, JAKs have a significant part in STATs phosphorylation. After dimerization of STATs, they translocate to the nucleus, where they either activate or suppress several target genes. This cascade is remarkably involved in the control of immune responses. Dysregulation of JAK-STAT signaling is associated with different immune disorders (82, 83). Besides, REG3A, acts as a key molecule for overexpression of the JAK2/STAT3 pathway which effectively contributes to triggering inflammation (84). b) The NF-kB canonical or classical signaling pathway is initiated from the cell surface receptor of pro-inflammatory cytokines and PAMPs containing TNFR, TLR and T/B cell receptor. The activation of IKK complex is triggered *via* binding of ligand molecules to transfer the signal across the cell surface. This complex generally comprises heterodimer of IKKα and IKKβ catalytic subunits and an IKKγ regulatory subunit. The released NF-kB dimers (most generally the p50–P65 dimer) could be translocated to the nucleus, and bind to DNA to trigger activation of the down-stream gene transcription (85–87). In addition, NF-κB signaling cascade could be regulated *via* TNFAIP3 through deubiquitinating TNFR1-RIP1, IL-1R/TLR4-TRAF6, and NOD2-RIP2 pathways (88). Moreover, canonical NF-kB cascade could be activated by various members of the TNFRSF including GITR, TNFR2, 4-1BB, and DR3 but not OX40 in Treg cells and modulates induction of Foxp3, markers of Th2/Th17 response (89). Mounting studies have revealed that multiple ncRNAs (lncRNAs and miRNAs) have an effective role in as major regulators of T cell function through regulating the JAK/STAT and NF-κB cascades. As an illustration, recent research has detected that downregulation of miR-128-3p could notably reduce the inflammation response of rheumatoid arthritis *via* attenuating the activity of NF-κB pathway and promote expression of TNFAIP3 (35). Another study has figured out that reducing the expression of lncRNA NEAT1 could lead to suppression of Th17/CD4+ T cell differentiation *via* downregulating STAT3 expression in rheumatoid arthritis patients (77). Furthermore, upregulation of DQ786243 could play a remarkable role in elevating the expression level of miR-146a through modulating Foxp3, and thereby suppressing the NF-κB signaling cascade in oral lichen planus disease (90). Green arrows indicate upregulation of target genes modulated *via* ncRNAs (lncRNAs, and miRNAs), red arrows depict inhibition by these ncRNAs. All the information regarding the role of these ncRNAs in modulating T call differentiation can be seen in **Tables 1**, **2**.

MALAT1 is another lncRNA with prominent role in the regulation of T cell function. This lncRNA regulates Th1/Th2 ratio by sponging miR-155 and modulating expression of CTLA4 (79). On the other hand, MEG3 has been found to enhance proportion of Th17 cells and regulate Treg/Th17 ratio by sponging miR-17 and upregulating RORγt (80). Moreover, this lncRNA decreases proliferation of CD4+T cell and inhibits Th1 and Th17 differentiation by absorbing miR-23a and modulating expression of TIGIT (81). **Figure 2** represents the role of various ncRNAs in regulating the JAK2/STAT3 and NF-κB signaling pathways in the regulation of function of T cells. **Table 2** summarizes the impact of lncRNAs on T cell function.

**Table 2:** LncRNAs and T cell regulation.

lncRNA	Expression pattern	Disease	Sample	Cell line	Interaction	Signaling pathway	Function	Reference
*IFNG-AS1*	Upregulation (in colonic tissues of IBD patients)	Inflammatory bowel diseases (IBD)	Colonic tissues from 11 IBD patients,	PBMCs from anonymous donors, Jurkat cells	–	–	Its overexpression augmented inflammatory cytokines expression and decrease anti-inflammatory cytokines expression in T cells.	(72)
*lnc-ITSN1-2*	Upregulation (in intestinal mucosa and PBMC of IBD patients)	Inflammatory bowel diseases (IBD)	Blood samples and intestinal mucosa specimens from 120 IBD patients and 30 healthy controls	CD4+ T Cells	miR-125a, IL-23R	–	Increased proliferation and activation of CD4+ T Cells and promoted their differentiation to Th1/Th17 by targeting miR-125a and upregulation of IL-23R	(73)
*lncRNA-CD160*	Upregulated (in CD8+ T cells of HBV infected patients)	Chronic hepatitis B virus (HBV) infection	Blood samples from 164 patients with chronic HBV infection and 67 healthy volunteers, C3H/HeN mice	CD160− CD8+ T cells and CD160+ CD8+ T cells	–	–	Decreased secretion of IFN-γ and TNF-α and repressed function of CD8+ T cells by recruiting HDAC11 to promoters of IFN-γ and TNF-α and elevating methylation of H3K9Me1	(91)
*NEAT1*	–	Sepsis	130 specific pathogen-free C57BL/6 male mice	CD4+ CD25+ T cells	miR-125, MCEMP1	–	Downregulation of NEAT1 has restricted immune response in mouse model of sepsis and induced T cell apoptosis through modulating miR-125/MCEMP1 axis	(76)
*NEAT1*	Upregulated (in CD4+ and CD8+ T cells of pSS patients)	Primary Sjögren’s syndrome (pSS)	Blood samples from 20 pSS patients and 10 healthy subjects	CD4+, CD8+ and CD19+ T cells, Jurkat cells	–	MAPK signaling pathway	Promoted expression of CXCL8 and TNF-α and activated MAPK signaling pathway	(34)
*NEAT1*	Upregulated (in the PBMCs of patients with RA)	Rheumatoid arthritis (RA)	Blood samples from 25 RA patients and 20 healthy controls, Male DBA/1J mice	CD4+ T cell	STAT3	–	Its silencing prevented differentiation of Th17 cells from CD4+ T cells by downregulating STAT3 through modulating its ubiquitination.	(77)
*NEAT1*	Upregulated (in PBMCs of HCC patients)	Hepatocellular carcinoma (HCC)	Blood samples from 20 HCC patients and 20 healthy controls	CD8+ T cells	miR-155, Tim-3↑	–	Its knockdown decreased apoptosis and raised cytotoxicity of CD8+ T cells by miR-155-mediated downregulation of Tim-3.	(78)
*lnc-EGFR*	Upregulated (in Treg cells of HCC patients)	Hepatocellular carcinoma (HCC)	Blood and tissue samples from 70 HCC patients and 55 healthy controls	CD4+ T cells, tumor infiltrated lymphocytes (TIL), 97H, Huh7	EGFR	–	Induced differentiation of Treg cells and impeded CTLs function through stabilizing EGFR by interfering with its ubiquitination	(92)
*PVT1*	Upregulated (in the CD4+T cells of patients with SS)	Sjögren’s syndrome (SS)	Blood samples and labial salivary gland tissues from SS patients and healthy controls, female C57BL/6 mice, NOD/ShiLtj mice and wild-type ICR mice	CD4+ T cell	Myc	–	Its downregulation decreased CD4+ T cells proliferation and impeded effector function in these cells through downregulation of Myc and controlling glycolysis	(93)
*lncRNA Morrbid*	–	Viral infection	C57BL/6 (WT), B6.SJL-Ptprc^a^ Pepc^b^/Boy (B6.SJL), and B6.129S1-Bcl2l11^tm1.1Ast/J^ (Bcl2l11 knock-out) mice, Ifnar1^tm1.1Ees^ (Ifnar1^flox^), TgCD4-Cre (CD4-cre), and Tg(TcrLCMV) (P14) mice	CD8+ T cells	–	PI3K-AKT signaling pathway	Regulates proliferation, survival and effector functions of CD8+ T cells by modulating Bcl2l11 expression and PI3K-AKT signaling pathway	(94)
*RP11-340F14.6*	Upregulated (in JIA patients)	Juvenile idiopathic arthritis (JIA)	Blood samples from JIA and healthy controls	T cell	P2X7R	–	Increased Th17 differentiation and inhibited Treg distribution by binding to P2X7R and inducing its expression	(95)
*MALAT1*	–	Asthma	Blood samples from 772 asthma patients and 441 healthy controls	CD4+ T cells	miR-155, CTLA4	–	Regulated Th1/Th2 ratio by sponging miR-155 and modulating expression of CTLA4	(79)
*MEG3*	Upregulated (in CD4 + T cells of patients with asthma)	Asthma	Blood samples from 52 asthma patients and 45 healthy controls	CD4 + T cells	miR-17↓, RORγt	–	Elevated proportion of Th17 cells and regulated Treg/Th17 ratio by sponging miR-17 and upregulating RORγt	(80)
*MEG3*	Downregulated (in CD4 + T cells of AA patients)	Aplastic anemia (AA)	Blood samples from 15 AA patients and 10 healthy controls	CD4+T cell	miR-23a, TIGIT	–	Its overexpression decreased proliferation of CD4+T cell and inhibited Th1 and Th17 differentiation by absorbing miR-23a and modulating expression of TIGIT	(81)
*DQ786243*	Upregulated (in CD4+ cells of OLP patients)	Oral lichen planus (OLP)	Blood samples from 10 OLP patients and 10 healthy volunteers	CD4+ T cell	miR-146a, Foxp3	NF-κB signaling pathway	Its overexpression increased Treg cells percentage and Foxp3 expression and promoted suppressive function of these cells by modulating Foxp3-miR-146a-NF-κB axis	(90)
*AW112010*	Upregulated (in activated CD4+ T cells)	–	Female C57BL/6J mice	CD4+ T cells	KDM5A	–	Induces differentiation of inflammatory T cells through inhibiting expression of IL-10 *via* interacting with KDM5A and histone demethylation	(96)
*GAS5*	Downregulated (in CD4+ T cells of HIV infected patients)	AIDS	Blood samples from 142 HIV infected patients and 58 healthy controls	CD4+ T cells	–	–	Regulated apoptosis rate and function of CD4+ T cells during HIV infection by modulating miR-21	(97)
*LINC00176*	Upregulated (in CD4+T cells of patients with SLE)	Systemic lupus erythematosus (SLE)	Blood samples from SLE patients and healthy individuals	CD4+ T cells	WIF1	WNT5a signaling pathway	Raised proliferation and adhesion of CD4+T cells by reducing WIF1 levels and WNT5a pathway activation	(98)
*lncRNA028466*	Downregulated (in CD4+ T cells of mice immunized with rEg.P29 antigen)	–	Female BALB/c mic	CD4+ T cell, CD8+ T cell	–	–	Implicated in regulation of cytokine expression from CD4+ T cells	(99)
*NONHSAT196558.1 (TANCR)*	–	–	Blood samples normal volunteers	Primary γδ T cells, Jurkat cells	TRAIL	–	Increased activation and cytotoxicity of γδ T cells by modulating expression of TRAIL in *cis* manner	(100)
*Dreg1*	–	–	Male C57BL/6 mice	splenic CD4+ T cells from mice and human	Gata3	–	Its expression was associated with expression of Gata3 during Th2 differentiation and regulated Gata3 expression during development of immune system	(101)
*Gm15575*	Upregulated (in Th17 cells and spleen tissues of EAE mice)	Multiple sclerosis (MS)	C57BL/6 mice	CD4+ T cells	miR-686, CCL7	–	Promoted Th17 differentiation by sponging miR-686 and upregulating expression of CCL7	(102)
*lncDDIT4*	Upregulated (in CD4+ T cells and PBMCs of patients with MS)	Multiple sclerosis (MS)	Blood samples from 36 MS patients and 26 healthy controls	naive CD4+ T cells	DDIT4	DDIT4/mTOR Pathway	Suppressed Th17 differentiation by targeting DDIT4 and inhibiting DDIT4/mTOR signaling	(103)
*linc-MAF-4*	Upregulated (in PBMCs of patients with MS)	Multiple sclerosis (MS)	Blood samples from 34 MS patients and 26 healthy subjects	Naive CD4+ T cells	MAF	–	Suppressed Th2 differentiation and promoted Th17 differentiation by inhibiting MAF expression	(104)
*NKILA*	Upregulated (in CTLs and TH1 cells of patients with breast and lung cancer)	Non-small cell lung cancer (NSCLC) and breast cancer	Tissue samples and blood samples from 576 h invasive breast carcinoma patients and 256 NSCLC patients, blood samples from healthy donors, NOD.SCID mice	CD8+ and CD4+ T cells, cytotoxic T lymphocyte (CTL), Th1, Th2 and Treg	NF-κB	–	Sensitized CTLs and Th1 cells to activation-induced cell death in tumor microenvironment and facilitated tumor immune evasion through suppression of NF-κB activity	(105)
*IFNA-AS1*	Downregulated (in PBMCs of patients with MG)	Myasthenia gravis (MG)	Blood samples from 32 MG patients and 20 healthy volunteers, female C57/BL6 mice	CD4+ T cell, Jurkat T cell	HLA-DRB1	–	Is implicated in regulation of Th1/Treg cell proliferation and activation of CD4 + T cells by influencing HLA-DRB1	(106)
*GATA3-AS1*	–	–	Blood samples from healthy volunteers	Human PBMC	GATA3	–	Regulated polarization of Th2 cells by increasing expression of GATA3	(107)

## CircRNAs and T Cell Regulation

CircRNAs are another group of ncRNAs that can be occasionally translated into proteins. The enclosed structure of circRNAs has endowed them a certain resistance to RNases and thus increases the stability in different body compartments (108). A genome wide transcriptome profiling of circRNAs has revealed that the median length of circRNAs is about 530 nt (109). Four categories of circRNA shave been identified: exonic circRNAs, circRNAs from introns, exon-intron circRNAs and intergenic circRNAs (110). The impact of this group of transcripts on T cell functions has been discovered during the recent decade. Several studies have shown that circRNAs can bind with miRNAs, thus decreasing bioavailability of miRNAs and releasing miRNA targets from their inhibitory effects. This kind of interactions between circRNAs and miRNAs has critical biological impacts. A high-throughput microarray study found down-regulation of circ_0001806 in patients with cryptococcal meningitis as compared to healthy controls. Circ_0001806 silencing has increased the intensity of fungal infection in the animal models and decreased their survival. Circ_0001806 has been suggested to regulate molecular cascades associated with the host antimicrobial response in T cells. Functionally, circ_0001806 has been shown to increase ADM level, decrease cell apoptosis and reverse G1/S arrest in T cells through sequestering miR-126. Thus, circRNA-1806/miR-126 cascade has an essential role in the regulation of cell cycle and apoptosis in T cells (111).

**Figure 3:**
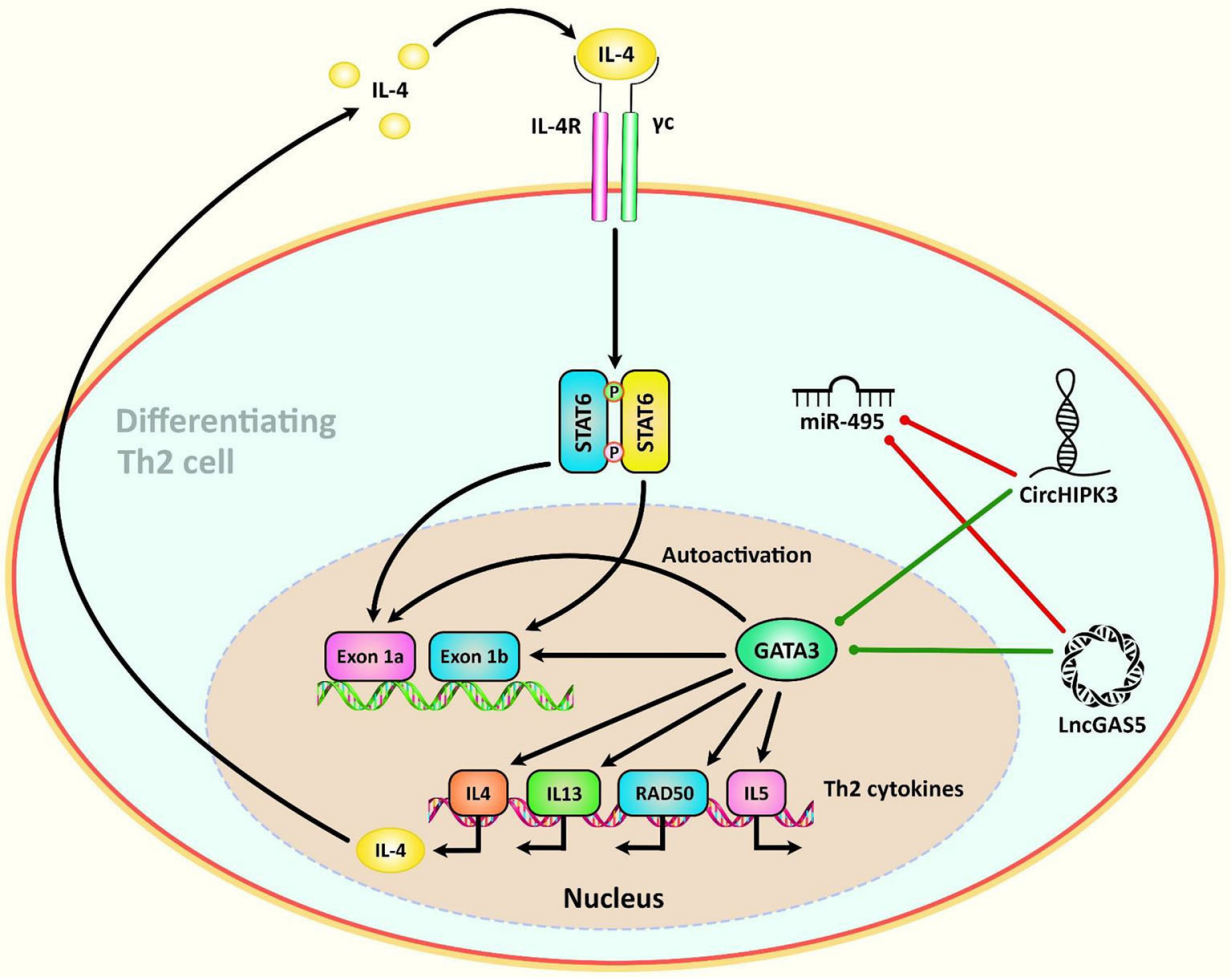
A schematic diagram of the role of some ncRNAs in modulating the IL-4-STAT6-GATA3 axis in Th2-cell differentiation. Th2 cell differentiation requires considerable metabolic reprogramming. Upon encountering cognate antigen in the lymph node, naive CD4 T helper cells are differentiated into Th2 cells under the effect of the IL-4-STAT6-GATA3 axis. GATA3 could, in turn, alter the IL4– IL13–IL5 locus to generate a conformation that is reachable by different other transcription factors that are involved in triggering the differentiation of T cells into T H2 cells (117). Growing evidence has confirmed that the interactions between CircHIPK3, LncGAS5, and miR-495 could play a crucial role in the modulation of Th2 differentiation in allergic rhinitis (116). Green arrows indicate upregulation of target genes by ncRNAs (lncRNA, and circRNA), red arrows depict inhibitory effects of by these ncRNAs.

Another high throughput circRNA profiling in patients with systemic lupus erythematosus (SLE) has led to identification of 127 differentially expressed circRNAs in T cells of these patients. Among them, circRNA hsa_circ_0045272 has been reported to be the down-regulated. Functional studies have shown that hsa_circ_0045272 silencing increases early apoptosis of Jurkat cells and enhances production of IL-2 by activated Jurkat cells. Binding of this circRNA with hsa-miR-6127 has been validated through functional studies (112). Hsa_circ_0012919 has been reported to be up-regulated in CD4+ T cells of SLE patients in two independent studies. In a microarray study of circRNAs signature in these patients, hsa_circ_0012919 has been among differentially expressed circRNAs between SLE patients and healthy subjects. Expression of this circRNA has been associated with SLE features. Down-regulation of hsa_circ_0012919 has enhanced expression of DNMT1, decreased CD70 and CD11a levels and inverted the DNA hypomethylation of these genes in CD4+ T cells of SLE. Hsa_circ_0012919 has been found to regulate expressions of KLF13 and RANTES through sequestering miR-125a (113). This circRNA has also been found to increase the expression of MDA5 in CD4+ T cells through downregulating miR-125a-3p (114). Hsa_circ_0005519 is another circRNA influencing T cell function. This circRNA has been found to be up-regulated in CD4+ T cells of asthmatic patients. Expression of this circRNA has been inversely correlated with hsa-let-7a-5p levels. Expression of hsa_circ_0005519 in CD4+ T cells has been correlated with fraction of exhaled nitric oxide and eosinophil ratio in the circulation of these patients. Hsa_circ_0005519 has been predicted to sequester hsa-let-7a-5p and release IL-13/IL-6 from its inhibitory effect (115). Being up-regulated circRNA in nasal mucosa of patients with allergic rhinitis, circHIPK3 has been found to promote differentiation of CD4+ T cells to Th2 by targeting miR-495 and increasing expression of GATA-3 (116). **Figure 3** illustrates the role of different ncRNAs in Th2-cell differentiation through modulating the IL-4-STAT6-GATA3 axis. **Table 3** shows the impact of circRNAs on T cell function.

**Table 3:** CircRNAs and T cell regulation.

circRNA	Expression pattern	Disease	Sample	Cell line	Interaction	Signaling pathway	Function	Reference
*circ_0001806*	Downregulated (in PBMCs of CM patients)	Cryptococcal meningitis (CM)	Blood samples from 20 CM patients and 18 healthy donors, female C57BL/6 mice	Jurkat T, CD3+ T cells	miR-126, ADM	–	Reduced apoptosis rate in T cells by sponging miRNA-126 and positive regulation of ADM	(111)
*hsa_circ_0045272*	Downregulated (in T cells of SLE patients)	Systemic lupus erythematosus (SLE)	Blood samples from 24 patients and 12 healthy controls	293T, Jurkat cells	hsa-miR‐6127	–	Its knockdown resulted in increased early apoptosis in T cells and elevated production of IL-2	(112)
*hsa_circ_0012919*	Upregulated (in CD4+ T cells of SLE patients)	SLE	Blood samples from 28 SLE patients and 18 healthy controls	CD4+ T cells	RNATES, KLF13, miR-125	–	Its downregulation led to upregulation of DNMT1 and decreased expression and hypermethylation of CD11a and CD70 in CD4+ T cells.	(113)
*hsa_circ_0012919*	Upregulated (in CD4+ T cells of SLE patients)	SLE	Blood samples from 20 SLE patients and 12 healthy controls	CD4+ T cells	miR-125a-3p, MDA5↑	–	Increases expression of MDA5 in CD4+ T cells through downregulating miR-125a-3p	(114)
*Hsa_circ_0005519*	Upregulated (in CD4+ T cells form patients with asthma)	Asthma	Blood samples from 65 asthmatic patients and 30 healthy individuals	CD4+ T cells	hsa-let-7a-5p	–	Augments expression of IL-13 and IL-6 by targeting hsa-let-7a-5p in CD4+ T cells	(115)
*CircHIPK3*	Upregulated (in nasal mucosal tissues of AR patients)	Allergic rhinitis (AR)	Blood samples and nasal mucosa specimens from 10 AR patients and 10 healthy controls, male BALB/c mice	CD4+ T cells	miR-495, GATA-3	–	Promotes differentiation of CD4+ T cells to Th2 by targeting miR-495 and increasing expression of GATA-3	(116)

## Summary

Numerous miRNAs, lncRNAs and circRNAs have been found to influence activity, survival or differentiation of T cells under physiologic or pathologic conditions. These molecules can affect pathophysiology of autoimmune conditions such as MS, SLE, RA, IBD and asthma *via* this route. Moreover, several of these non-coding RNAs influence immune evasion of cancer cells and their response to immunotherapeutic modalities.

Notably, both lncRNAs and circRNAs can serve as sponges for miRNAs. Through this molecular mechanism, lncRNAs and circRNAs bind with certain miRNAs to decrease their bioavailability. Thus, circRNAs and lncRNAs can indirectly affect expression of miRNAs targets. Circ_0001806/miR-126, hsa_circ_0045272/hsa-miR‐6127, hsa_circ_0012919/miR-125, hsa_circ_0005519/hsa-let-7a-5p, circHIPK3/miR-495 are examples of circRNA/miRNA axes regulating T cell functions. In addition, lnc-ITSN1-2/miR-125a, NEAT1/miR-125, NEAT1/miR-155, MALAT1/miR-155, MEG3/miR-17, MEG3/miR-23a, Gm15575/miR-686 are examples of lncRNA/miRNA pairs in this regard. These examples not only indicate the intricate network between these classes of transcripts, but also provide clues to find the most important modules in the regulation of T cell functions. Contribution of miR-125, miR-155, MEG3 and NEAT1 in more than one of these molecular axes suggests their significance in the regulation of T cell function. Most notably, all of these four non-coding RNAs have essential roles in cancer development or suppression (118–120), further highlighting the intercalation of cancer-related and immune-related molecular pathways.

GATA3, RORγt, NF-κB, SIRT1, STAT3 and FOXO3 as major regulators of T cell function have been shown to be influenced by non-coding RNAs. For instance, GATA3 is influenced by Dreg1 and GATA3-AS1 lncRNAs; RORγt is regulated by MEG3; SIRT1 is modulated by miR-155 and miR-23a-3p; STAT3 is regulated by let-7f-5p, miR-182-5p and miR-10a-3p, miR-21-5p and NEAT1; and FOXO3 is controlled by miR-155. Therefore, non-coding RNAs affect T cells functions through different routes.

Consistent with the important roles of lncRNAs, circRNAs and miRNAs in the regulation of function of T cells and their impact on differentiation of different classes of T cells, therapeutic targeting of these ncRNAs represent an efficient tool for management of disorders related with abnormal function of T cells. Forced up-regulation or silencing of these transcript can affect signaling pathways that modulate T cell responses, thus alleviating tissue damage caused by abnormal T cell responses. Moreover, assessment of ncRNAs signature is a probable strategy for prediction of course of T cell-related disorders.

Taken together, the significant impact of non-coding RNAs on differentiation, survival, cytokine production and activity of T cells potentiates these molecules as important targets for treatment of various disorders, particularly cancer. Moreover, non-coding RNAs participate in the pathogenesis of autoimmune disorders *via* affecting epigenetic regulation of genes with crucial roles in the regulation of effector T cells as well as Tregs (121). Thus, identification of the role of these transcripts in the regulation of T cell functions can provide new modalities for treatment of this kind of disorders as well. High throughput sequencing method and assessment of the competing endogenous RNA network through bioinformatics methods is an efficient strategy in identification of appropriate targets for therapeutic interventions.

## Future Perspectives

High throughput sequencing strategies and identification of differential expressions of lncRNAs, circRNAs, miRNAs and mRNAs in different stages of T cells development would help in recognition of role of each transcript in development of this group of blood cells. Further knock-in and knock-out studies in different disease conditions can help in identification of specific treatment strategies for related disorders.

## Author Contributions

SG-F, DB, and JK wrote the draft and revised it. MT and MP designed and supervised the study. OR and MT designed the figures and tables. All authors contributed to the article and approved the submitted version.

## Conflict of Interest

The authors declare that the research was conducted in the absence of any commercial or financial relationships that could be construed as a potential conflict of interest.

## Publisher’s Note

All claims expressed in this article are solely those of the authors and do not necessarily represent those of their affiliated organizations, or those of the publisher, the editors and the reviewers. Any product that may be evaluated in this article, or claim that may be made by its manufacturer, is not guaranteed or endorsed by the publisher.
